# Effect of Biobased Cling Films on Cheese Quality: Color and Aroma Analysis for Sustainable Food Packaging

**DOI:** 10.3390/foods12193672

**Published:** 2023-10-06

**Authors:** Chiara Chirilli, Luisa Torri

**Affiliations:** University of Gastronomic Sciences, Piazza Vittorio Emanuele II 9, 12042 Pollenzo, Italy; c.chirilli@unisg.it

**Keywords:** sustainable packaging, biobased material, electronic nose analysis, image analysis, consumer perception

## Abstract

Biobased and biodegradable polymeric materials are a sustainable alternative to the conventional plastics used in food packaging. This study investigated the possible effect of biobased cling films derived from renewable and circular and sustainable sources on key cheese sensory parameters (appearance and odor) able to influence consumer acceptance or rejection of a food product over time. For this purpose, a semi-hard cheese was selected as food model and stored for 14 days at 5 °C wrapped with five cling films: two bio-plastic materials from renewable circular and sustainable sources (R-BP1 and R-BP2), one bio-plastic film from a non-renewable source (NR-BP), and two conventional cling films (LDPE and PVC). Three analytical approaches (image analysis, electronic nose, and sensory test) were applied to evaluate the variation and the acceptability in terms of appearance and odor of the cheese. In preserving cheese color, the R-BP1 and RBP2 films were comparable to LDPE film, while NR-BP film was comparable to PVC film. In terms of odor preservation, R-BP2 film was comparable to LDPE and PVC. The consumer test showed that appearance and odor scores were higher for cheeses stored in R-BP1 and R-BP2 films than NR-BP film. Moreover, in terms of odor, R-BP1 film performed better than conventional films. This study shows how biodegradable cling films from renewable circular and sustainable resources could have comparable performance to conventional plastics (LDPE and PVC) used in the food sector.

## 1. Introduction

The environmental concern has promoted the urgency of developing sustainable materials for biodegradable and/or compostable food packaging [1,2]. Indeed, there is a growing interest in packaging made from renewable natural resources, and biobased polymers may be a viable substitute for synthetic plastics due to their ability to biodegrade and the availability of raw materials [3,4], as evidenced by the study by Chirilli et al. [5] who investigated how aware and attentive consumers were to concepts related to the environmental sustainability of food packaging.

Biobased plastics made from renewable resources are supposed to be less environmentally impactful and may represent a great opportunity in the food packaging industry. Reducing and repurposing waste materials, such as industrial by-products and secondary flows from industries, agricultural wastes, food wastes, and lignocellulosic materials, and using them as raw materials can help develop a sustainable circular bioeconomy system that would reduce the use of nonrenewable resources by promoting an environmentally friendly resource flow instead [6]. Therefore, the design and development of sustainable packaging is currently focusing not only on improving the performance of biopolymers and synthesizing new biobased and biodegradable materials but also on finding renewable sources, particularly undervalued raw materials, and implementing material reuse and recycling systems [7].

Among the biodegradable biopolymers that have found greater application in food packaging are cellulose, starch, polylactide (PLA) (a synthetic polyester produced from lactic acid) and polyhydroxyalkanoates (PHAs) (polyesters produced by microorganisms from carbohydrates or lipids), chitosan, soy protein isolate, whey protein isolate, and gluten [8]. Moreover, biobased materials are capable, like conventional packaging, of ensuring food safety and sensory quality by protecting food from moisture, oxygen, lipid oxidation, and loss of volatile components [9,10,11]. Overall, there are multiple benefits of using biobased materials manufactured from renewable and recyclable sources, as they reduce environmental impact by reducing carbon dioxide emissions, increase consumers’ environmental awareness, preserve barrier properties, ensure adequate food shelf life, and increase the sustainability of the products for which they are used [12]. Despite the various positive contributions that these materials make, packaging still mainly consists of polymers such PE, PET, PP, PS, PVC, and PA [13], probably because the latter offer higher performance, better processing, and lower overall costs [14]. Indeed, to prevent the occurrence of enzymatic, chemical, physical, and microbiological changes that result in spoilage reactions in food, appropriate packaging materials and methods should be chosen [15]. Biobased packaging materials must be able to maintain their barrier and mechanical properties in the condition of use and fulfill their preservation role until disposal [16]. 

Among the most commonly used types of food packaging materials, cling films (or cling wraps) play an important role. These films are generally disposable, which results in the production of large volumes of waste with a considerable effect on the environment. Cling films originated as thin, transparent plastic films employed to wrap food and maintain it for a given storage period. Because traditional synthetic polymers used for packaging are not biodegradable and have a negative impact on the environment, there are many studies on the development of cling films based on natural polymers, generally obtained by combining polysaccharides, including cellulose, chitosan, starch, pectin, alginate, and carrageenan, with lipids or proteins [17,18]. They are fully degraded by microorganisms to water, carbon dioxide, and biomass [19], and they are able to protect food from microbial spoilage, allow adequate storage, and maintain suitable barriers to oxygen and water vapor and good mechanical properties [2]. Piscopo et al. [20] studied a multilayer film composed of PLA coated with a silicon oxide barrier as a method of cheese packaging that proved to be a viable alternative to conventional plastic multilayer films, while Bharathi et al. [21] developed a biodegradable zein-based cling film and obtained good results comparing it with films consisting of synthetic polymeric materials.

In this context, it is therefore important to study solutions that can promote the use of biodegradable cling films for food packaging in order to reduce the production and consumption of disposable materials that have a negative impact on the environment. At the same time, it is essential to verify the consumers’ sensory acceptance for the food products wrapped with biodegradable cling films. Indeed, several studies [22,23,24,25] revealed that sensory appeal is a key factor influencing consumer perception, purchasing behavior, and satisfaction. In particular, appearance and odor are the first components of sensory appeal considered for the acceptability of a product, before tasting it. In fact, food appearance, influenced by surface color, is the first sensation that consumers perceive and take into account to accept or reject a food [23]. After appearance, odor is the second sensory parameter considered, because not only it helps to understand whether changes have occurred in the product, but also because if the orthonasal olfactory perception is unacceptable, very rarely will the product be tasted by consumers [24]. Therefore, the aim of this work was to evaluate how experimental biodegradable and compostable cling films obtained from renewable sources may have influenced the appearance and odor of cheese and its acceptability during storage, in comparison with biodegradable and compostable cling films obtained from non-renewable sources and those made of the most commercially used synthetic polymers (PVC and LDPE). Thus, the variations in appearance and odor of cheese packaged in the different biobased materials and fossil-based plastics were estimated during storage by combining instrumental techniques (image analysis and electronic nose) and sensory methods (consumer acceptability testing).

## 2. Materials and Methods

### 2.1. Cling Films

Five samples of cling films for food applications were analyzed. In particular, two innovative and experimental bio-plastic materials obtained from renewable sources (R-BP1 and R-BP2) were compared to one commercial bio-plastic film obtained from non-renewable source (NR-BP) and two commercial and conventional cling films (LDPE and PVC). The physical-chemical properties of the cling films are reported in Table 1. R-BP1 and R-BP2 were developed within the context of the “PRIME—Innovative Processes and Products of Green Chemistry” research project (https://www.unisg.it/en/ricerca/prime-innovative-processes-and-products-of-green-chemistry/, accessed on 28 August 2023), while NR-BP, LDPE, and PVC cling films are the commercial references selected as a benchmark and provided by the PRIME research project coordinators. The NR-BP, R-BP1, and R-BP2 cling films consist of biodegradable and compostable biopolyesters synthesized via polycondensation of aliphatic and/or aromatic dicarboxylic acids and diols. In particular, under the PRIME project and following the principles of the circular economy, the two innovative and experimental bio-plastic cling films from renewable sources are based on acids obtained from vegetable oils recovered from waste biomass present in Piedmont. Specifically, the biobased formulations R-BP1 and R-BP2 are composed of two polyesters synthesized from the biobased monomers azelaic acid and butanediol. The two cling films differ in the percentage of the monomers. Moreover, R-BP1 has a higher melting point than R-BP2 in order to ensure greater stability during the filming process, while R-BP2 has a lower crystallization temperature in order to optimize clinging capacity.

### 2.2. Cheese

A semi-hard cheese was chosen for this study because cheeses belonging to this category were previously used in experimental studies aimed to test cling film packaging [26,27]. In particular, the semi-hard Bra PDO cheese was selected as the food product to compare the effects of the selected cling films on the preservation of food quality during storage because several reasons: (i) it is usually bought or stored at home packaged in cling films; (ii) it has a uniform internal paste in terms of color and appearance (suitable for image analysis); (iii) it has a quite intense odor (suitable for electronic nose analysis); (iv) it is easy to cut into uniform shaped and sized slices (suitable for sensory tests); (v) it is not expensive; (vi) it is produced in the town where the present study was conducted, thus being a local product could be considered a socially and environmentally sustainable choice. The Bra PDO cheese was purchased at a local cheese store (Bra, Italy). According to D.P.R. 54/97 specifications, the Bra PDO cheese is a semi-fat, pressed cheese made from cow’s milk, possibly with slight additions of sheep’s and/or goat’s milk. The color is white or ivory white and the texture of the paste is moderately consistent and elastic, with very small, barely visible, and not too diffused holes. The flavor is pleasantly aromatic, moderately piquant and savory. The percentage of fat in dry matter is a minimum of 32 per cent. Aging period is a minimum of forty-five days. The Bra PDO cheese was cut into 21 slices (≈250 g each), 124 slices (≈10 g), and 560 slices (≈15 g) for image analysis, electronic nose analysis, and sensory evaluation, respectively. Cheese slices were individually wrapped in the five different cling films and stored for 14 days at 5 ± 1 °C. The study was stopped after 14 days, when some mold appeared on the surface of the cheese, thus making unsuitable the cheese for the acceptability test.

### 2.3. Image Analysis

The color variation of cheese wrapped in the five cling films after 0, 4, 7, 9, 10, 11, and 14 days of storage time (T0, T4, T7, T9, T10, T11, T14) was assessed by image analysis. For each film type, three cheese slices (3 replicates) were analyzed at each control time, for a total of 21 slices. At each control storage time, the three replicates of slices of cheese were unwrapped and placed on black cardboard, and images were acquired with a digital camera in jpeg format. After the analysis, the three replicates were rewrapped with the cling film and stored at 5 ± 1 °C. The camera was placed perpendicularly to the sample under controlled lighting conditions. The lighting system consisted of one cool daylight lamp also placed perpendicularly above the sample to avoid irregular reflection on the sample surface. Images were elaborated by the ImageJ version 1.51 software to obtain the intensity mean values of R (red), B (blue), and G (green) color indices. These values were converted as L*, a*, and b* coordinates (CIE L*a*b* color space) with EasyRGB (www.easyrgb.com/en/convert.php, accessed on 8 August 2022) and used to calculate the hue (a/b) and saturation (a^2^+b^2^)^1/2^ indexes [28]. 

### 2.4. Electronic Nose

The variation in odor of cheese wrapped in the five cling films at different storage times (T4, T7, T9, T10, T11, T14) was evaluated. For each film type, four cheese slices (four replicates) were analyzed at each control time, for a total of 120 slices. These samples were compared with the aroma profile of the cheese at T0 (four replicates). The total number of slices analyzed was thus 124. The emissions of volatile compounds were assessed by the portable PEN 3 e-nose (Win Muster Airsense Analytic Inc., Schwerim, Germany) equipped with 10 metal oxide semiconductors (MOS) and fully described by Torri et al. [29]. Cheese (10.0 ± 1 g) was placed in 45 mL glass air-tight vials and hermetically sealed with a PTFE/silicone septum and a screw cap. The vials were stored at −18 ± 1 °C until analysis. Then, the vials were equilibrated at 25 ± 1 °C for 1 h and analyzed at the same temperature under standardized conditions. The measurement device aspirated the gaseous compounds from the headspace of the sample through the sensor array at 400 mL/min for 30 s. After sample analysis, the system was purged for 70 s at a flowrate of 600 mL/min with filtered air prior to the injection of the next sample to allow the instrument to re-establish a baseline. Four replications were performed for each sample (124 measurements in total), and the average of the sensor responses was used for the subsequent statistical analysis.

### 2.5. Sensory Evaluation

Fifty-six untrained subjects (M = 20, F = 36; mean age = 26, age range = 20–55; Italians = 37, non-Italians = 19) were recruited among staff and students of the University of Gastronomic Sciences (Bra, Italy). Subjects voluntarily joined the sensory test and signed an informed consent before starting the test. The study was approved by the Ethics Committee of the University of Gastronomic Sciences (Ethics Committee Proceeding no. 2022.01) and conducted according to the guidelines of the Declaration of Helsinki.

Consumers’ acceptability for the appearance and odor of the cheese wrapped in the cling films was assessed after 7 and 10 days of storage (T7 and T10, respectively). Since the cling films tested were still experimental and migration data capable of ensuring the safety of tasters were not available, it was deemed appropriate not to evaluate the level of acceptability for taste but to evaluate only the acceptability of appearance and smell of the packaged samples. In order to minimize the possible subjective biases associated with the sensory evaluation due to physiological and psychological factors [30], a standardized procedure of sample presentation and evaluation was adopted. In particular, thirty minutes before the sensory test, cheese samples were taken out of the fridge and kept at room temperature (25 ± 1 °C) until evaluation. One slice of cheese for each type of cling film was unwrapped and put in a 90 cc PLA container sealed with parafilm and codified with three-digit codes. Samples were served in a randomized order across subjects. Participants were instructed to observe and odor the sample but to not taste it, then to rate how much they disliked/liked the appearance and the odor on a nine-point hedonic scale (1 = extremely dislike, 9 = extremely like). A 30 s waiting procedure was enforced between samples. Evaluation was conducted in individual booths under white light. The experimenters verbally introduced the consumers to the computerized data collection procedure (FIZZ Acquisition software, version 2.46A, Biosystèmes, Courtenon, France). The consumers completed their evaluation in approximately 15–20 min. 

### 2.6. Data Analysis

The values of the color parameters obtained from the cheese sample wrapped in each cling film at each storage time were subtracted from the color values observed for its reference sample (T0), in order to calculate the single delta values for L* (ΔL), a* (Δa), b* (Δb), hue (Δ hue), and saturation (Δ saturation) indexes and for the total color difference (ΔE). 

The matrices of the single delta values obtained for each cling film sample were independently submitted to one-way analysis of variance (ANOVA) models applied to determine the main effects of storage time on the color evolution of sample. Moreover, the matrices of the delta values obtained for each cling film sample were independently submitted to one-way analysis of variance (ANOVA) models applied to determine the overall main effects of cling film (fixed factor; five levels: R-BP1, R-BP2, NR-BP, PVC, and LDPE) and storage time (fixed factor; seven levels: T0, T4, T7, T9, T10, T11, T14) on color parameters (ΔE, ΔL, Δa, Δb, Δ hue, and Δ saturation). The output of the ANOVA models provided the overall mean delta values of the color parameters as a function of both each cling film and storage time. Additionally, the combined effect of the material used for wrapping and the storage time in relation to color parameters (ΔE, ΔL, Δa, Δb, Δ hue, and Δ saturation) was analyzed by means of principal component analysis (PCA). 

Concerning the e-nose data, responses from the 10 MOS sensors were submitted to principal PCA to evaluate the variation over time of the aromatic profile of cheese packaged in the five different cling films. 

Regarding the sensory data, two-way ANOVA mixed models (fixed factors: variables material and time of storage; random factor: subject) were independently applied to investigate the effect of the cling film and storage time on appearance and odor acceptability of cheese. Subjects’ preferences regarding the appearance and odor of the products packaged in the different cling films at the two storage times considered were also submitted to PCA to obtain an internal preference map. 

All ANOVA models were followed by Tukey’s HSD (honestly significant difference) test. Significance criteria were set at alpha equal to 0.05 for all analyses. All analyses were conducted using the XLSTAT statistical software package version 2021.2.2 (Addinsoft, New York, NY, USA).

## 3. Results and Discussion

### 3.1. Effect of the Cling Films on Cheese Color over Storage Time

The one-way ANOVA models applied to the delta values of the color parameters to analyze the effect of the cling film on the variation in appearance over time permitted the delineation of the gradual trend in the variation of the color parameters over the storage period, identifying the times at which the main variations occur (Table 2). Total color difference ΔE, as the main quality indicator, can be used to represent the overall color change [31], while ΔHue is usually used as a ripeness indicator for visualizing the color of food products effectively [32]. In addition, the hue parameter is among the most discriminating parameters in visual comparison because it is able to be perceived well by the human eye [33]. In the present study, it was found that the color parameters examined varied depending on the film material considered, in accordance with previous research [34,35,36]. The parameters that varied the most over time for all cling films were Δa and Δ hue. In particular, for the cheese packaged with NR-BP, there are significant differences for the parameters delta a (*p* < 0.0001) and delta hue (*p* < 0.0001), whereby after seven days of storage, the values of both parameters start to increase, resulting in the cheese being significantly different from that stored for 4 days. The Δa and Δhue parameter values remain stable until T14, whereby the highest values for both parameters were found to be significantly different from the other control times. On the other hand, for cheese packaged with R-BP1, the parameters that present significant differences as a function of their variation over time were ΔE (*p* = 0.007) and Δhue (*p* = 0.021), whose significant variations occured between T4 and T14. The cheeses packaged with R-BP2 and PVC showed similar variations over time; specifically, significant differences were found for the parameters Δa (*p* = 0.020 for R-BP2 and 0.0004 for PVC) and Δ hue (*p* = 0.009 and *p* = 0.002, for R-BP2 and PVC, respectively), with the times that were significantly different between them being T4 and T14. With regard to cheeses packaged with LDPE, the parameters with the greatest significant differences were ΔE (*p* = 0.001), Δa (*p* = 0.022), Δb (*p* = 0.019), and Δ hue (*p* = 0.012). For all parameters, the main variations and significant differences were found between T4 and T14.

Specifically, to provide an overview of the effects of cling films over the entire storage period and the effect of all times on the variation of color parameters, the one-way ANOVA models applied to color delta mean values showed a significant effect of the different cling film used on ΔE, ΔL, Δa, and Δ hue parameters, while no significant differences were observed for Δb and Δ saturation (Table 3). The observed color variations could be explained taking into account the oxygen and water vapor barrier properties of the cling films. In particular, concerning the ΔE parameter, the highest mean value was observed for the cheese wrapped in NR-BP film that was significantly different (*p* = 0.002) from the cheese wrapped in R-BP1, PVC, and LDPE cling films. The resulted color differences could be partly determined also by the different WVTR values characterizing the three cling films, since the NR-BP film has higher WVTR values than the films made from renewable and circular resources, R-BP1 and R-BP2. In fact, higher water vapor permeability may affect shelf life and may lead to a variation in food quality, thus influencing weight loss and color variation [37]. In the case of cheese, a_w_ is responsible for its stability. In particular, water activity depends mainly on moisture and salt content, so the rate of water vapor transmission through the packaging material is crucial in controlling a_w_ [38]. These results are in agreement with the study by Peixoto et al. [39] in which different ΔE values were found over time between cheese packaged in fossil-based plastic and cheese packaged in biodegradable packaging made from renewable resources in which color changes led to darkening of the cheese, explained by the fact that lower water activity due to the removal of water from the cheese surface probably caused Maillard reactions between lactose and lysine residues of the proteins. Significant differences were found for the ΔL parameter (*p* < 0.0001), with cheese wrapped in R-BP1 and LDPE film having higher values than cheese wrapped in NR-BP and PVC films. The slices of cheese packaged with R-PB1 and R-BP2 showed significant differences (*p* = 0.010) to the slices of cheese packaged with LDPE films regarding the Δa parameter. Only the cheese wrapped in PVC and LDPE films was significantly different for the Δ hue parameter (*p* = 0.027). The clear difference between the behavior of LDPE cling films and PVC cling films could be attributable to a different oxygen transmission rate that distinguish the two types of material (6738 ± 1002 and 3253 ± 554 cm^3^ m^−2^ 24 h^−1^ bar^−1^, respectively), confirming what reported in several studies [40,41]. Oxygen permeability is the parameter that could have most influenced the cheese evolution. In fact, exposure of cheese to oxygen can trigger enzymatic oxidation reactions resulting in degradation processes such as discoloration, production of off-flavors, loss of nutrients, and formation of toxic substances. Regarding the main effects of storage times on color parameters, significant differences were found for Δa and Δ hue (*p* < 0.0001) where the cheese stored for 14 days had the highest mean value, while the cheese stored for four days turned out to have a lower value. The only significant difference due to Δb was found for the cheese stored for 14 days with the lowest value. Although no particular significant differences were found in Δb values but only in Δa values, the combined parameter hue may lead to some insights. Carotenoids are the compounds that give the yellow color. Therefore, less oxidation of carotenoids due to a higher oxygen barrier may have helped to maintain a more yellow color, resulting in less variation in hue [32]. This study agrees with this statement because cheeses packaged in PVC, which has the highest oxygen barrier, experienced less hue variation than cheeses packaged in LDPE.

Furthermore, despite the different WVTR values between the LDPE cling film, characterized by the lowest values of water vapor permeability, and the cling films R-BP1 and R-BP2, there do not seem to be any great differences in the color analysis of the samples packaged with these films. Probably, in this case, these results could be attributable to a balancing of both WVTR and O_2_TR values, such that the LDPE films present higher values than the R-BP1 and R-BP2 films, confirming that, although biological and biodegradable materials have a higher water vapor permeability than fossil-based plastics, they are still characterized by relatively good gas barrier properties [2,42]. On the contrary, the one-way ANOVA did not show significant differences for ΔE, ΔL, and Δ saturation.

**Figure 1:**
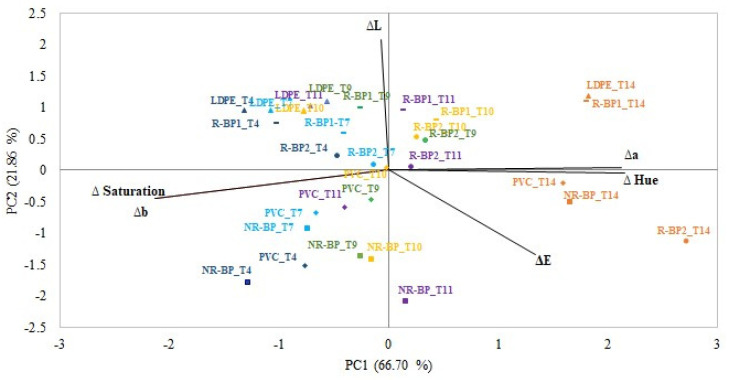
Biplot from the principal component analysis applied to interaction of the cling film and the storage time in relation to color parameters (ΔE, ΔL, Δa, Δb, Δ hue, and Δ saturation). Different symbols identify different cling film materials, while different colors identify different storage times.

The biplot obtained from the PCA applied to the interaction of the material used for wrapping and the storage time in relation to color parameters is shown in Figure 1. The two first principal components (PC1 and PC2) accounted for 88.57% of the total variance at 66.70% and 21.86%, respectively. The score plot, which illustrates the mutual relationships between samples and color parameters, shows the sample separation according to the storage condition. Indeed, samples were distributed along PC1 and PC2 according to the cling film and storage time, respectively. In particular, according to the distribution of the samples along the PC1, there is a clear separation among the cling films, implying a similarity in performance and in the variation of the color parameters between the NR-BP and PVC packaged cheese samples, which are distinguished from the cheese samples packaged in the other three cling films (R-BP1, R-BP2, and LDPE). The similar trend in Δa variation between cheese packaged in PVC and in NR-BP cling film is partially in contrast to the study of Brandelero et al. [43] in which the lettuce stored in PVC presented lower luminosity (L) and a higher value of the b* parameter than that stored in biodegradable films. In addition, the distribution of materials in the biplot could be due to oxygen barrier values similar for the cling films R-BP1 (5050 ± 837 cm^3^ m^−2^ 24 h^−1^ bar^−1^), R-BP2 (6235 ± 947 cm^3^ m^−2^ 24 h^−1^ bar^−1^), and LDPE (6738 ± 1002 cm^3^ m^−2^ 24 h^−1^ bar^−1^), but higher than that of the PVC cling film (3253 ± 554 cm^3^ m^−2^ 24 h^−1^ bar^−1^). The change in color is correlated with the carotenoid content variation [44]. Therefore, higher oxygen permeability could have led to degradation of carotenoids and consequently to color fading [33]. This could confirm the results obtained in this study in which samples packaged with the lowest oxygen barrier materials (LDPE, R-BP1, and R-BP2) experienced a greater change in lightness. A difference in lightness may be due to a denaturation of ß-lactoglobulin and its conjugation to j-casein [33]. Moreover, the second component discriminates samples according to their storage time: taking into consideration that color is considered as an explanatory factor for deterioration [31], on the right-hand side of the biplot are the samples that have been stored the longest. This would indicate that higher values of Δb, Δ saturation, and ΔL describe the samples during the first days of storage. In contrast, higher values of Δa, Δ hue, and ΔE mainly describe the samples at the end of the storage.

### 3.2. Effect of the Cling Films on Cheese Aroma Profile over Storage Time

**Figure 2:**
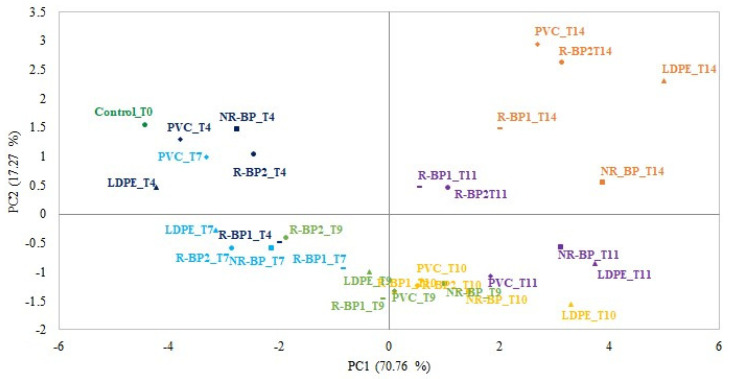
Biplot from the principal component analysis applied to e-nose responses. Different colors identify different storage times.

The biplot of the PCA applied to the e-nose data is shown in Figure 2. The two first principal components, PC1 and PC2, accounted for 88.03% of the total variance at 70.76% and 17.27%, respectively. The first PC discriminates samples according to their storage time, outlining the trend in the aroma profile of wrapped cheese samples. The aromatic profiles of the cheese slices packaged in the different cling films appear to have a similar evolution over time. During the first control times (T4 and T7), the cheese wrapped with R-BP1 seemed to have a slower decay than the cheeses packaged with the other cling films, while at the end of the storage period (14 days), the cheeses packaged with R-BP1 cling film seemed to have a slightly faster decay, similar to the cheeses packaged with NR-BP. At day 14 of storage, the cheeses wrapped with R-BP2 showed a comparable development of the aromatic profile to the cheeses wrapped with the commercial reference cling films PVC and LDPE. These results could be due to the fact that these two cling films have higher WVTR values, compared to both fossil-based plastics cling films (PVC and LDPE), and higher O_2_TR values compared to PVC cling film. Thus, lower water vapor and oxygen permeability barriers could be responsible of a lower cheese sensory shelf life [45]. A high degree of oxygen diffusion from the surrounding atmosphere in cheeses and thus increased oxygen in packages can in fact cause rancidity and other oxidative degradation reactions in lipid-containing foods, leading not only to a possible risk of reduced quality and safety, but also to the production and spread of unpleasant and undesirable odors that can spread outside the package as well [46,47]. The similar oxygen permeability values, instead, of the R-BP2 film with the LDPE cling film could confirm the greater similarity in the development of the aroma profile at the end of storage. 

### 3.3. Effect of the Cling Films on Acceptability for Cheese Appearance and Odor over Storage Time

The mean values of the scores provided by all subjects regarding the acceptability evaluation of appearance and odor of the slices of cheese packaged into the five cling films are reported in Table 4. In terms of appearance, both cling film materials and different storage times had a significant effect on the subjects’ judgement. In fact, subjects preferred cheese wrapped in R-BP1 and R-BP2 cling films that resulted in significant difference (*p* = 0.005) with the cheese wrapped in NR-BP film. Slices of cheese wrapped into PVC and LDPE films were not significantly different from the cheese wrapped with the other three types of cling films. Regarding storage times, the subjects distinguished between the two storage times and preferred cheese stored for 7 days (*p* = 0.026). Conversely, in terms of odor, there was not a significant difference between the two storage times. However, concerning the effect of material on odor acceptability, significant differences were found (*p* = 0.0003). Subjects showed a higher acceptability for R-BP1 cling film packaged cheese, while the lowest mean score was achieved for cheese packaged in NR-BP film. R-BP2 packaged cheese was not found to be significantly different from either cheese wrapped with R-BP1 or cheese wrapped with PVC and LDPE, but there was a significant difference with the NR-BP packaged cheese.

**Figure 3:**
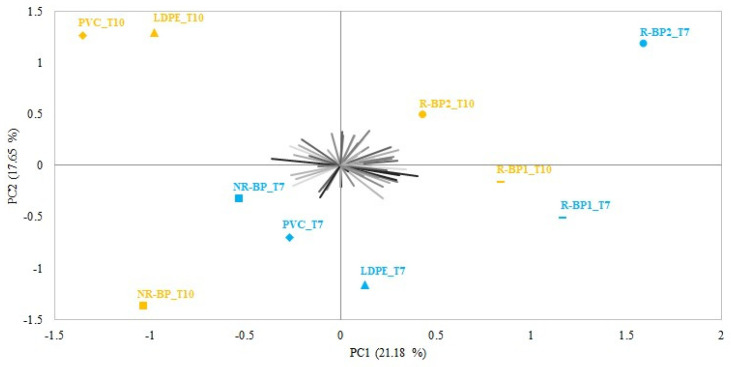
Biplot from the principal component analysis applied to acceptability data for cheese appearance (n = 56). Different symbols identify the two different storage times (T7 and T10).

The preference map obtained from the PCA applied to the appearance acceptability data expressed by the subjects during the sensory evaluation is shown in Figure 3. The total variance explained for the samples based on the first two significant dimensions was 38.83%, with PC1 and PC2 accounting for 21.18% and 17.65%, respectively. Samples were distributed along the first component PC1 mainly according to the type of cling film, showing a clear difference between cheeses wrapped with R-BP1 and R-BP2 from the samples wrapped with the other types (NR-BP, PVC, and LDPE). Instead, samples tended to be distributed along the second component PC2 as a function of storage time. According to time, there was a similarity between cheeses packaged with R-BP1 and R-BP2 films, and it was evident that cheeses packaged with R-BP1 and R-BP2, even with longer storage times, were appreciated more than cheeses with shorter storage times packaged into NR-BP, PVC, and LDPE cling films by a higher proportion of subjects (identified by the vectors in Figure 3). In fact, the visual distribution of the subjects’ acceptability seems to lean more towards cheeses packaged with R-BP1 and R-BP2 films and stored for 7 and 10 days.

The results obtained from the sensory tests, according to which cheese packaged with the R-BP1 and R-BP2 coating films exhibited higher acceptability in terms of appearance, are partially in disagreement with the results of the instrumental tests applied in the present study, which would instead seem to show a higher decay of some color parameters. However, the sensory tests showed that cheese packed with the NR-BP cling film was less liked by consumers in terms of appearance, which could validate the results obtained from the instrumental tests, which showed that cheese packed with NR-BP differs the most from the control.

**Figure 4:**
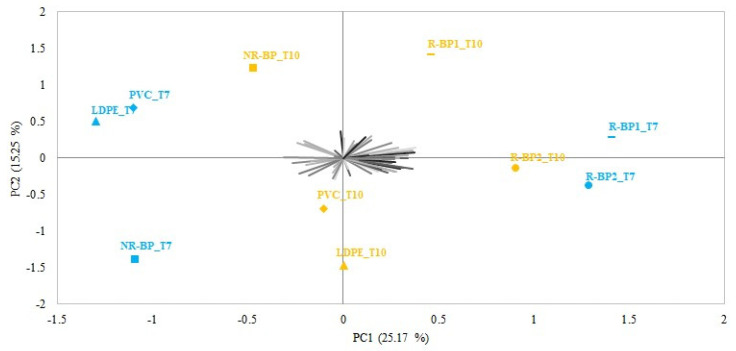
Biplot from the principal component analysis applied to acceptability data for cheese odor (n = 56). Different symbols identify the two different storage times (T7 and T10).

The total variance explained by the preference map obtained for the evaluation of the odor of the stored cheese by PCA applied to the acceptability data expressed by the subjects during the sensory evaluation was 40.2%, with PC1 and PC2 accounting for 25.17% and 15.25%, respectively (Figure 4). Even this preference map seems to confirm the subjects’ preferences for cheese stored with the cling films R-BP1 and R-BP2. Indeed, the visual distribution of acceptability response is more oriented to the slices of cheese wrapped into R-BP1 and R-BP2 cling films, also indicating in the case of odor acceptability, a similarity of appreciation between these two cling films, whereas these two samples were quite clearly distinguished from the cheese stored in the LDPE, PVC, and NR-BP cling films. This partially disagrees with previous studies according to which foods packaged with biodegradable films were less appreciated than those packaged with conventional packaging. In fact, cheese packaged with the R-BP1 cling film was more appreciated in terms of odor by the participants than cheese packaged with the others cling films, except for R-BP2. That is the case of the work of Pluta-Kubica et al. [37], in which the odor of the cheese wrapped in the active biodegradable film was less appreciated by the panelists than that of the control, possibly as a consequence of the decrease in water content following a much higher WVTR of the biodegradable film. Amjadi et al. [48] also observed lower hedonic odor scores and overall acceptance during storage of white cheese wrapped in biodegradable films containing zinc oxide nanoparticles.

The fact that this study only examined appearance and odor variation during the storage period of packaged products emerged as a limitation, since the variations in terms of flavor and texture that food products may be subjected to during storage could not be identified [49,50,51,52,53]. Therefore, future research could envisage tests including all sensory modalities. Moreover, other analyses (e.g., weight loss monitoring, fat oxidation analyses, microbiological analyses, pH measurements, and rheological analyses) on wrapped cheese would be helpful in order to provide a more comprehensive evaluation of the preservation performance offered by the films analyzed. It could also be interesting in the future to analyze the effects of these cling films on other types of cheese (e.g., hard cheese), dairy products, and food products, such as fruit and vegetables, in order to assess whether the films may have different effects when in contact with different food matrices.

## 4. Conclusions

This study examined the potential of biobased and biodegradable cling films derived from renewable and circular sources in preserving cheese appearance and odor, comparing them with commercially cling films derived from non-renewable sources and with fossil-based plastic cling films, in order to reduce the future production and consumption of disposable food contact materials that have a negative impact on the environment. Based on the determination of cheese color variation and odor profile development, the results showed a different protecting performance of the packaging materials as a function of storage time and cling film type. Specifically, according to the results obtained from instrumental analysis, cheeses packaged in films derived from renewable and nonrenewable sources were found to be more prone to color variation than cheeses packaged in PVC and LDPE, probably due to different water vapor and oxygen transmission rate values. However, when analyzing the variation over time, comparable performances were found between R-BP1 and R-BP2 films and LDPE films, while biodegradable film derived from nonrenewable sources showed more similarities with PVC film. Regarding odor evolution, although similarities were found between the biodegradable and synthetic films, the samples packed with NR-BP and R-BP1 seemed to have slightly faster decay in the last days of storage, unlike the PVC-packed cheese, which showed a slower decay. Overall, this study shows how the biobased and biodegradable cling films tested, obtained from renewable and circular sources, have a potential to be comparable solutions to conventional plastic materials. Therefore, future research could be addressed to improving these innovative films able to offer multiple benefits for sustainable development.

## Figures and Tables

**Table 1:** Properties of the cling films used.

Code	From Renewable and Cicrular Source	Biodegradable and Compostable Material	Thickness (mm)	WVTR ^a^ (ASTM F1249, 23 °C, 50% RH, g m^−2^ 24 h^−1^)	O_2_TR (ASTM D3985, 23 °C, 50% RH, cm^3^ m^−2^ 24 h^−1^ bar^−1^)	Tensile Properties					Puncture Test		
						(ASTM D882, 23 °C, 50% RH, V_0_ 50 mm/min)					(ASTM D5748, 23 °C, 50% RH- V_0_ 250 mm/min)		
						Direction	sb (Mpa)	eb (%)	E (Mpa)	Enb (kJ/m^2^)	Fmax (N)	Enb (mJ)	Stroke @Fmax (mm)
R-BP1	Yes	Yes	20 ± 1	77 ± 8	5050 ± 837	MD	53 ± 2	343 ± 16	123 ± 15	5649 ± 349	31 ± 2	89 ± 3	90 ± 3
						TD	36 ± 3	649 ± 39	125 ± 12	5038 ± 298			
R-BP2	Yes	Yes	20 ± 3	90 ± 11	6235 ± 947	MD	42 ± 2	356 ± 17	87 ± 8	4923 ± 320	29 ± 3	84 ± 2	102 ± 4
						TD	30 ± 2	625 ± 35	94 ± 7	4476 ± 365			
NR-BP	No	Yes	20 ± 3	125 ± 13	5022 ± 1066	MD	66 ± 3	322 ± 15	119 ± 10	5679 ± 371	36 ± 3	121 ± 3	98 ± 3
						TD	53 ± 4	593 ± 42	158 ± 17	5752 ± 496			
PVC	No	No	20 ± 1	39 ± 5	3253 ± 554	MD	26 ± 1	163 ± 7	76 ± 6	1390 ± 71	45 ± 3	83 ± 4	78 ± 2
						TD	32 ± 2	246 ± 13	99 ± 8	2233 ± 107			
LDPE	No	No	20 ± 2	1.0 ± 0.2	6738 ± 1002	MD	40 ±3	393 ± 18	231 ± 19	4129 ± 289	20 ± 2	91 ± 3	95 ± 1
						TD	22 ±2	734 ± 51	270 ± 23	4124 ± 301			

^a^ Relative value compared to the value obtained for LDPE. sb: strain at break; eb: elongation at break; E: elastic modulus; Enb: 5-ethylidene-2-norbornene content; Fmax: maximum force to puncture; MD: machine direction; TD: transverse direction.

**Table 2:** Color parameters of cheese samples wrapped in the five tested cling films for different storage times.

Cling Film	Storage Time	ΔE	ΔL	Δa	Δb	Δ Hue	Δ Saturation
R-BP1	T4	**3.9 ^b^**	1.6 ^a^	−0.2 ^a^	0.03 ^a^	**−0.01 ^b^**	0.03 ^a^
	T7	**4.1 ^b^**	0.6 ^a^	0.7 ^a^	−0.5 ^a^	**0.03 ^ab^**	−0.5 ^a^
	T9	**3.9 ^b^**	2.3 ^a^	0.9 ^a^	−0.9 ^a^	**0.03 ^ab^**	−0.9 ^a^
	T10	**4.9 ^b^**	1.1 ^a^	1.3 ^a^	−3.1 ^a^	**0.1 ^ab^**	−3.1 ^a^
	T11	**3.3 ^ab^**	0.8 ^a^	1.3 ^a^	−2.2 ^a^	**0.1 ^ab^**	−2.2 ^a^
	T14	**9.1 ^a^**	5.1 ^a^	2.3 ^a^	−6.1 ^a^	**0.1 ^a^**	−6.1 ^a^
*p*		**0.007**	0.416	0.104	0.227	**0.021**	0.291
R-BP2	T4	6.8 ^a^	1.3 ^a^	**0.2 ^b^**	−0.7 ^a^	**0.01 ^b^**	−0.7 ^a^
	T7	7.5 ^a^	0.7 ^a^	**0.5 ^b^**	−1.6 ^a^	**0.02 ^b^**	−1.7 ^a^
	T9	6.9 ^a^	1.7 ^a^	**1.2 ^ab^**	−2.4 ^a^	**0.05 ^b^**	−2.4 ^a^
	T10	6.1 ^a^	1.0 ^a^	**1.1 ^ab^**	−2.7 ^a^	**0.04 ^b^**	−2.8 ^a^
	T11	6.3 ^a^	−1.2 ^a^	**1.0 ^ab^**	−2.6 ^a^	**0.04 ^b^**	−2.7 ^a^
	T14	12.5 ^a^	−5.3 ^a^	**2.3 ^a^**	−10.3 ^a^	**0.1 ^a^**	−10.3 ^a^
*p*		0.557	0.647	**0.020**	0.522	**0.0004**	0.528
NR-BP	T4	8.6 ^a^	−5.5 ^a^	**−0.5 ^c^**	2.9 ^a^	**0.02 ^c^**	2.9 ^a^
	T7	7.9 ^a^	−2.1 ^a^	**0.4 ^b^**	2.3 ^a^	**0.02 ^b^**	2.3 ^a^
	T9	9.2 ^a^	−3.5 ^a^	**0.9 ^b^**	1.6 ^a^	**0.04 ^b^**	1.6 ^a^
	T10	9.9 ^a^	−4.1 ^a^	**0.5 ^b^**	−0.4 ^a^	**0.02 ^b^**	−0.4 ^a^
	T11	11.0 ^a^	−6.2 ^a^	**1.1 ^b^**	0.3 ^a^	**0.1 ^b^**	0.3 ^a^
	T14	9.1 ^a^	−2.8 ^a^	**2.0 ^a^**	−5.5 ^a^	**0.1 ^a^**	−5.4 ^a^
*p*		0.990	0.995	**<0.0001**	0.393	**<0.0001**	0.401
PVC	T4	6.9 ^a^	−6.4 ^a^	**0.2 ^b^**	1.5 ^a^	**0.01 ^b^**	1.5 ^a^
	T7	4.8 ^a^	−4.3 ^a^	**0.5 ^b^**	1.1 ^a^	**0.02 ^b^**	1.1 ^a^
	T9	5.2 ^a^	−3.6 ^a^	**1.0 ^ab^**	−0.2 ^a^	**0.05 ^b^**	−0.2 ^a^
	T10	4.1 ^a^	−2.4 ^a^	**1.2 ^ab^**	−0.9 ^a^	**0.1 ^b^**	−1.0 ^a^
	T11	4.8 ^a^	−4.2 ^a^	**0.8 ^ab^**	0.3 ^a^	**0.03 ^ab^**	0.3 ^a^
	T14	7.3 ^a^	−3.1 ^a^	**2.1 ^a^**	−5.8 ^a^	**0.1 ^a^**	−5.7 ^a^
*p*		0.724	0.722	**0.009**	0.103	**0.002**	0.108
LDPE	T4	**3.7 ^b^**	2.7 ^a^	**−0.6 ^b^**	**0.7 ^a^**	**−0.02 ^b^**	**0.8 ^a^**
	T7	**3.8 ^b^**	3.1 ^a^	**−0.02 ^ab^**	**1.3 ^a^**	**0.002 ^b^**	**1.3 ^a^**
	T9	**4.7 ^b^**	3.5 ^a^	**0.2 ^ab^**	**0.4 ^a^**	**0.01 ^ab^**	**0.4 ^a^**
	T10	**6.1 ^ab^**	5.5 ^a^	**0.4 ^ab^**	**0.4 ^a^**	**0.02 ^b^**	**0.3 ^a^**
	T11	**4.3 ^b^**	3.7 ^a^	**0.4 ^ab^**	**0.7 ^a^**	**0.02 ^ab^**	**0.6 ^a^**
	T14	**9.7 ^a^**	5.3 ^a^	**1.8 ^a^**	**−7.5 ^b^**	**0.1 ^a^**	**−7.5 ^b^**
*p*		**0.001**	0.036	**0.022**	**0.019**	**0.012**	**0.019**

Text in bold and different lowercase letters in each column indicate statistically significant differences between mean values related to cheese wrapped in the same cling film material and stored for different storage times (Tukey’s HSD test, *p* < 0.05).

**Table 3:** Overall mean delta values of the color parameters as a function of both each cling film and storage time.

Variables	ΔE	ΔL	Δa	Δb	Δ Hue	Δ Saturation
*Cling film ^a^*						
R-BP1	**4.9 ^b^**	**1.9 ^a^**	**1.0 ^a^**	−2.1 ^a^	**0.04 ^ab^**	−2.2 ^a^
R-BP2	**7.7 ^ab^**	**−0.3 ^ab^**	**1.1 ^a^**	−3.4 ^a^	**0.04 ^ab^**	−3.4 ^a^
NR-BP	**9.3 ^a^**	**−4.0 ^b^**	**0.7 ^ab^**	0.2 ^a^	**0.04 ^ab^**	0.2 ^a^
PVC	**5.5 ^b^**	**−4.0 ^b^**	**1.0 ^ab^**	−0.7 ^a^	**0.05 ^a^**	−0.7 ^a^
LDPE	**5.4 ^b^**	**4.0 ^a^**	**0.4 ^b^**	−0.7 ^a^	**0.02 ^b^**	−0.7 ^a^
*p*	**0.002**	**<0.0001**	**0.010**	0.110	**0.027**	0.107
*Storage time ^b^*						
T4	**6.0 ^a^**	−1.3 ^a^	**−0.2 ^c^**	**0.9 ^a^**	**−0.01 ^c^**	**0.9 ^a^**
T7	**5.6 ^a^**	−0.4 ^a^	**0.4 ^bc^**	**0.5 ^a^**	**0.02 ^bc^**	**0.5 ^a^**
T9	**6.3 ^a^**	0.5 ^a^	**0.9 ^b^**	**−0.3 ^a^**	**0.04 ^b^**	**−0.3 ^a^**
T10	**5.9 ^a^**	−0.2 ^a^	**0.8 ^b^**	**−1.4 ^a^**	**0.04 ^b^**	**−1.4 ^a^**
T11	**5.9 ^a^**	−1.4 ^a^	**0.9 ^b^**	**−0.7 ^a^**	**0.04 ^b^**	**−0.7 ^a^**
T14	**9.5 ^a^**	−0.2 ^a^	**2.1 ^a^**	**−7.1 ^b^**	**0.11 ^a^**	**−7.0 ^b^**
*p*	**0.045**	0.932	**<0.0001**	**<0.0001**	**<0.0001**	**<0.0001**

Mean values in bold and different lowercase letters in each column indicate statistically significant differences (Tukey’s HSD test, *p* < 0.05).

**Table 4:** Acceptability mean values for cheese appearance and odor (n = 56).

Variables	Appearance	Odor
*Cling film*		
R-BP1	**5.7 ^a^**	**5.3 ^a^**
R-BP2	**5.6 ^a^**	**5.1 ^ab^**
NR-BP	**5.1 ^b^**	**4.6 ^c^**
PVC	**5.4 ^ab^**	**4.6 ^bc^**
LDPE	**5.6 ^ab^**	**4.7 ^bc^**
*p*	**0.005**	**0.0003**
*Storage time*		
T7	**5.6 ^a^**	4.9 ^a^
T10	**5.3 ^b^**	4.8 ^a^
*p*	**0.026**	0.776

Mean values in bold and different lowercase letters within a column indicate statistically significant differences in terms of appearance and odor acceptability (Tukey’s HSD test, *p* < 0.05).

## Data Availability

The data presented in this study are available on request from the corresponding author. The data are not publicly available due to privacy.
